# The potential of microdialysis to estimate rifampicin concentrations in the lung of guinea pigs

**DOI:** 10.1371/journal.pone.0245922

**Published:** 2021-01-22

**Authors:** Faye Lanni, Neil Burton, Debbie Harris, Susan Fotheringham, Simon Clark, Oliver Skinner, Nathan Wiblin, Mike Dennis, Stuart Armstrong, Geraint Davies, Ann Williams

**Affiliations:** 1 Public Health England, Salisbury, Wiltshire, United Kingdom; 2 Q3 Analytical, Porton Science Park Incubator Centre, Salisbury, United Kingdom; 3 DSTL, Salisbury, United Kingdom; 4 Clinical Infection, Microbiology and Immunology, University of Liverpool, Brownlow Hill, Liverpool, United Kingdom; Institut de Pharmacologie et de Biologie Structurale, FRANCE

## Abstract

Optimised pre-clinical models are required for TB drug development to better predict the pharmacokinetics of anti-tuberculosis (anti-TB) drugs to shorten the time taken for novel drugs and combinations to be approved for clinical trial. Microdialysis can be used to measure unbound drug concentrations in awake freely moving animals in order to describe the pharmacokinetics of drugs in the organs as a continuous sampling technique. The aim of this work was to develop and optimise the microdialysis methodology in guinea pigs to better understand the pharmacokinetics of rifampicin in the lung. *In vitro* experiments were performed before progressing into *in vivo* studies because the recovery (concentration of the drug in the tissue fluid related to that in the collected dialysate) of rifampicin was dependent on a variety of experimental conditions. Mass spectrometry of the dialysate was used to determine the impact of flow rate, perfusion fluid and the molecular weight cut-off and membrane length of probes on the recovery of rifampicin at physiologically relevant concentrations. Following determination of probe efficiency and identification of a correlation between rifampicin concentrations in the lung and skeletal muscle, experiments were conducted to measure rifampicin in the sacrospinalis of guinea pigs using microdialysis. Lung concentrations of rifampicin were estimated from the rifampicin concentrations measured in the sacrospinalis. These studies suggest the potential usefulness of the microdialysis methodology to determine drug concentrations of selected anti-TB drugs to support new TB drug development.

## Introduction

Tuberculosis (TB) drug development is a slow and often unsuccessful process, requiring a minimum of six years for a novel TB drug and at least 20 years for a novel regimen to be approved for use in the clinic [1]. This is one of the reasons why TB is still a major global health problem with 1.2 million deaths in 2018 alone [2]. There are numerous factors that contribute to the pace of drug development including the slow growth of mycobacteria, the complicated spectrum of TB disease in humans, including latent infection and a lack of well-validated models and tools for pre-clinical development, especially those that encompass the range of physiological states of the bacteria (i.e. latency). It has become apparent that in order to shorten the time taken for regulatory approval of novel anti-TB drugs there is a need for predictive pre-clinical models. In particular, *in vivo* models that allow measurement of drug concentrations at more clinically relevant sites are required because such measurement in humans is rarely feasible. The development of *in vivo* models that enable quantitation of drugs in organs would enable a better understanding of the relationship between drug concentrations in the blood (traditional site of measurement) and concentrations within the biophase of infection. These models are particularly important when the site of infection is at a location that is not easily accessible, such as the granuloma [3]. Additionally there is a growing number of studies [4] as well as encouragement from the U.S Food and Drug Administration (FDA) to support monitoring of drug concentrations in tissues to ensure adequate drug concentrations in order to suppress antimicrobial resistance and treatment failures [5].

Microdialysis is one of the few techniques that allows continuous quantification of drug(s) in organs, over time, in freely moving animals thus enabling the pharmacokinetic profiles of drugs in organs to be determined. The continuous nature of the sampling is due to the fact that microdialysis does not interfere with blood homeostasis [6].

Guinea pigs are an important pre-clinical model for TB because, following aerosol infection, disease progression closely recapitulates several features of human disease including well-organised granulomas with central regions of necrosis [7]. A number of studies have demonstrated the utility of this model for pre-clinical evaluations of novel TB drug candidates or regimens [7–11]. Given the potential of microdialysis as a tool to generate continuous samples from an individual and to measure drug concentrations in more relevant sites than blood, we sought to investigate the feasibility of this technique in guinea pigs to measure organ concentrations of the front-line anti-TB drugs rifampicin and isoniazid. As microdialysis of the lungs is technically difficult in a freely-moving animal and a correlation between drug concentrations in the lungs and skeletal muscle had been previously reported [12, 13], it was hypothesised that dialysis of the sacrospinalis (skeletal muscle along spine) could be used as a surrogate for lung [14].

*In vitro* microdialysis experiments were conducted before progressing to *in vivo* experiments in order to determine the relative recovery (concentration of the drug in the organ related to that in the collected dialysate) which is dependent upon a variety of experimental and environmental conditions [6]. The impact of flow rate, perfusion fluid, probe characteristics (molecular weight cut-off and membrane length) and organ type (lung and skeletal muscle) on the relative recovery of physiologically relevant drug concentrations were investigated. The experiments that determined the relative recovery of the probes were repeated three times (as a minimum) to enable a mean relative recovery to be calculated and to obtain an error on these measurements. The recoveries of the replicate experiments were expanded to determine the efficiency (and associated error) of the probe. Subsequently, the drug concentrations measured following *in vivo* microdialysis were corrected for using this calculated probe efficiency.

## Materials and methods

### Animals

Specific pathogen free, out-bred female Dunkin-Hartley guinea pigs (weighing between 200 and 250g) were obtained from accredited breeders/suppliers and were housed in compliance with the UK Home Office Code of Practice for the Housing and Care of Animals Bred, Supplied or Used for Scientific Purposes (2014). All experimental procedures were conducted under the authority of a project licence approved by the UK Home Office after being reviewed by Public Health England, Porton Down local animal welfare and ethical review body (AWERB).

### Determination of drug concentrations in lung and skeletal muscle

Guinea pigs were administered one oral dose of isoniazid (50mg/kg) or rifampicin (50mg/kg) in a fruit puree. Guinea pigs were necropsied either 60, 120 or 240 minutes post drug administration to remove lung and skeletal muscle. Samples were processed via protein precipitation [15] and drug concentrations were determined via LC-MS/MS.

### Microdialysis method and calculation of relative recovery

For all *in vitro* microdialysis experiments, a CMA 402 syringe pump (CMA Microdialysis AB, Sweden) was fitted with a 1ml syringe that contained perfusion fluid. The syringe was connected to a CMA 20 microdialysis probe which was placed in either a rifampicin or isoniazid containing solution (drug (10μg/ml) + HPLC water) with or without the addition of lung or skeletal muscle homogenate. In total four different probes were evaluated, each had either a 4mm or 10mm membrane length with two different molecular weight cut-offs (20kDa or 100kDa), probes that had been re-used following previous exposure to the drug (rifampicin) after storage in demineralised water were also evaluated. Three different perfusion fluids; T1 (CMA Microdialysis AB, Sweden), CNS (CMA Microdialysis AB, Sweden) and Ringers Solution and four different flow rates (1, 2, 5 &10μl/minute) were tested. For *in vivo* microdialysis experiments, an additional 20cm of sterile FEP tubing was used to attach the probe to the liquid swivel. Samples were collected every 30 minutes for 240 minutes. The collected dialysate was analysed via LC-MS/MS.

Relative recovery was calculated using the following equation:

$$Relative\;Recovery\;(\%)=\left(\frac{C_{dialysate}}{C_{periprobe}}\right)\times 100$$

*C*_*dialysate*_ is the concentration of drug in dialysate, *C*_*periprobe*_ is the concentration of drug in the fluid surrounding the probe (the drug solution in which the microdialysis probe was submerged/ drug in the skeletal muscle).

The triplicate determinations of the relative recoveries at different drug concentrations (10μg/ml to 1mg/ml) were expanded to determine the efficiency (and associated error) of the probe [16]. Errors were expanded using standard error of the mean of relative recoveries. The equation used to calculate error was:

$$\surd \;{\left(\text{a}\right)}^{2}+{\left(\text{b}\right)}^{2}+\ldots \ldots .+{\left(\text{z}\right)}^{2}$$

The error was then divided by mean %relative recovery to determine the fractional uncertainty.

### Proof of concept *in vivo* microdialysis study

Surgery was conducted on the guinea pigs under anaesthesia (isoflurane (2–2.5%/0.4–0.8L/min oxygen)) for probe implantation into the sacrospinalis [6, 7]. Tissue glue was used to seal the wound prior to placing the guinea pig in a harness (CIH95 Infusion Harness for Rat; CMA Microdialysis) ensuring that there was no strain on the probe and tubing exiting the sacrospinalis. The spring that exited the harness containing the tubing was attached via a liquid swivel to a counter-balanced lever arm which was fitted to a modified guinea pig cage. Approximately two hours post-surgical completion, a rifampicin (10μg/ml) flush was conducted to ensure probe patency and limit non-specific binding. This flush was then repeated three times during the recovery period. Guinea pigs were administered (pre- and post- operatively) both pain relief and anti-inflammatories (buprenorphine (0.03mg/kg) and carprofen (4mg/kg) respectively). Throughout this recovery period the guinea pigs were constantly observed remotely via a camera and twice- daily health checks were performed to monitor wound recovery and harness tightness. Guinea pigs were dialysed on day 3 post-surgery following an oral drug administration of a single 50mg/kg rifampicin dose, following a one hour pre-flush with warmed T1 perfusion fluid (non-rifampicin containing) to ensure that the rifampicin measured in the collected fractions were not an artefact of the *in situ* rifampicin flush. Microdialysis samples were collected every 30 minutes for 240 minutes. Upon completion, lungs and skeletal muscle were removed and samples prepared for LC-MS/MS as described in Kjellsson et al. [15].

### LC-MS/MS

Individual stock solutions of rifampicin and isoniazid were prepared in dimethyl sulfoxide (DMSO) and further diluted using 0.1% formic acid. 10μl of 1μg/ml dextromethorphan (DXT) was added to calibration solutions and samples as the internal standard. Calibration standards were prepared by spiking T1 perfusion fluid with known amounts of rifampicin or isoniazid over a concentration range of 2000, 1000, 500, 250, 100, 50, 25, 10, 5 and 1ng/ml. The HPLC system consisted of a Sciex Agilent 1100 pump, column heater and degasser connected to a CTC HTS auto-sampler. Chromatographic separation of rifampicin was achieved using a Kinetex C8 50 x 2.1mm, 5μm column. Chromatographic separation of isoniazid was achieved using a Kinetex C8 150 x 2.1mm, 5μm column. Rifampicin and DXT were eluted using a mobile phase composed of 0.1% formic acid aqueous (A) and 0.1% formic acid in acetonitrile (B) according to the following gradient programme: 0 minute 95% A, 1 minute 95% A, 4 minute 5% A, 5 minute 5% A, 5.1minute 95% A, 8 minute 5% A. Isoniazid and DXT were eluted using a mobile phase composed of 0.1% formic acid aqueous (A) and 0.1% formic acid in acetonitrile (B) according to the following gradient programme: 0 minute 95% A, 5 minute 95% A, 10 minute 5% A, 15 minute 5% A, 15.1minute 95% A, 20 minute 95% A. The mass spectrometer was an API 3000 with an electrospray source operating in positive mode. Nebuliser gas was 14PSI, curtain gas 14PSI, CAD gas 4PSI, ion-spray Voltage (IS) 5500V and ion source temperature 350°C. Multiple reaction monitoring (MRM) mode was used for quantitation using transitions 823.4 → 95.3 for rifampicin, 137.9 → 120.9 for isoniazid and 272.4 → 215.2 for DXT. Peak areas were integrated using Analyst 1.4 software.

## Results

### Correlation between drug concentrations in lung and skeletal muscle

Total isoniazid concentrations were measured in the lungs and sacrospinalis removed (post mortem) from guinea pigs given a single dose of 50mg/kg isoniazid, 1, 2 or 4 hours post dosing. The isoniazid concentrations in the lung and skeletal muscle were not the same and therefore the concentrations in the two organs were analysed to identify if there was a relationship or correlation between them that would enable the concentration in the lung to be derived from the skeletal muscle. The analysis revealed that there was not a relationship between lung and skeletal muscle drug concentrations in animals dosed with isoniazid (Pearson R^2^ value = 0.1857 (P = 0.3936)).

**Fig 1:**
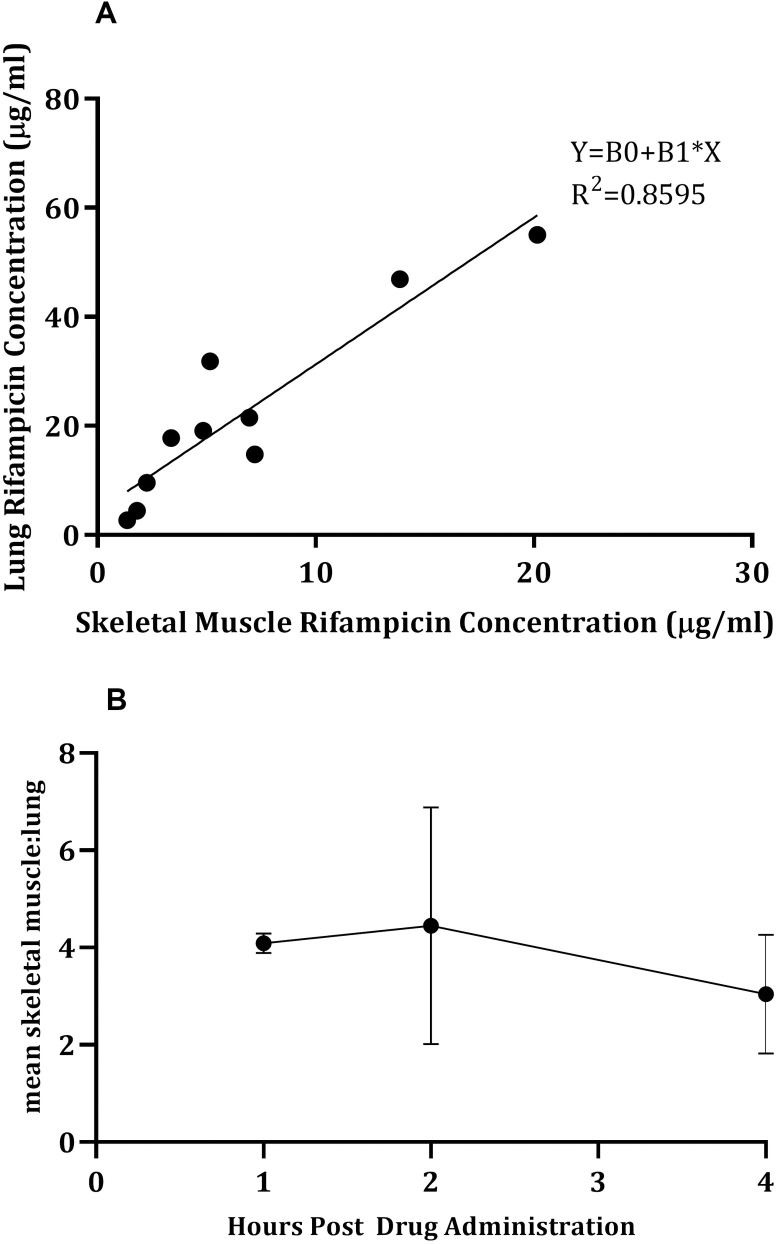
**A**: Correlation between rifampicin concentrations measured in lung and skeletal muscle. Data fitted with linear interpolation and the following equation generated; Y = B0+B1*X, where B0 = 4.317, B1 = 2.695, X = unbound rifampicin concentration (μg/ml) recovered from sacrospinalis can be used to calculate Y, the estimated unbound rifampicin concentration in lung (μg/ml). **B**: Mean ratio of rifampicin concentrations between lung and skeletal muscle plotted over time. Each point is the mean data of multiple guinea pigs (n =), which were culled 1 (n = 2), 2 (n = 4) or 4 (n = 6) hours post drug administration. Error bars represent the standard deviation.

Total rifampicin concentrations were measured in the lungs and sacrospinalis removed (post mortem) from guinea pigs given a single dose of 50mg/kg rifampicin, 1, 2 or 4 hours post dosing. A correlation (Pearson R^2^ = 0.8595 (P value = 0.0001)) was identified between the two tissue types. Linear interpolation was used to fit the data (Sy.x = 6.91) (Fig 1A) and the subsequent equation, Y = B0+B1*X, could be used to calculate the estimated unbound concentration of rifampicin in the lungs (Y), based on the unbound rifampicin concentration (μg/ml) measured in the sacrospinalis (X), (B0 = 4.317 (±3.381) and B1 = 2.695 (±0.38)) (equation constants generated using GraphPad Prism)).

To understand if the time at which the samples were collected (60, 120, 240 minutes) had an effect on the relationship between drug concentrations in the lung and skeletal muscle, the ratio of rifampicin concentrations between lung and skeletal muscle were plotted over time (Fig 1B). Although the ratio showed variation, especially at 120 minutes which may be explained by differing distribution kinetics in out-bred guinea pigs, these data indicated that the relationship of drug concentrations between the lung and skeletal muscle was relatively consistent regardless of sampling time point. These data provided confirmation that the sacrospinalis was a potentially suitable tissue to dialyse in order to estimate concentrations of rifampicin in the lung. Rifampicin, rather than isoniazid, was therefore selected for proof of concept *in vivo* microdialysis studies using the sacrospinalis for probe insertion with the assumption that this relationship (between lung and skeletal muscle rifampicin concentrations) would remain true for unbound rifampicin concentrations as collected via microdialysis.

### *In vitro* optimisation

A number of different CMA 20 probes were trialled in order to achieve a consistent relative recovery at physiologically relevant rifampicin concentrations (10μg/ml) with and without the presence of organ (lung and skeletal muscle) homogenate. Two different molecular weight cut-offs of the probe membrane were tested and the relative recovery for the higher molecular weight cut-off (100kDa) was marginally higher (~1%) than that of the lower molecular weight cut-off (20kDa) in a drug solution that contained no proteins. Probes that had been re-used following previous exposure to rifampicin [19] (after storage in demineralised water), had a higher relative recovery (16.3%) and the results were less variable (mean %CoV 35%) than probes that had not been previously exposed to rifampicin (relative recovery 8.8%, mean %CoV 58%). This increased relative recovery associated with the probe which had been previously exposed to rifampicin was expected because of the lipophilic nature of rifampicin. Relative recovery was further increased when a previously exposed probe with a 10mm membrane length (as opposed to 4mm membrane length) was used. Therefore, the probe selected for *in vivo* microdialysis was a 10mm membrane with a 20kDa molecular weight cut-off that had previously been exposed to rifampicin. A flow rate of 2μl/ minute was selected as adequate rifampicin recovery and sufficient sample volume for analysis were achieved. T1 perfusion fluid was selected for use after a trial of a number of perfusion fluids as its use contributed to consistent relative recoveries. The calculated probe efficiency of the selected probe was 21±9% and the rifampicin concentrations measured following *in vivo* microdialysis were corrected for using the calculated probe efficiency (21±9%). A probe efficiency greater than 20% is sufficient [17], as this would recover rifampicin at concentrations above the lower limit of quantitation (LLOQ) for LC-MS/MS analysis (5ng/ml).

### *In vivo* microdialysis

**Fig 2:**
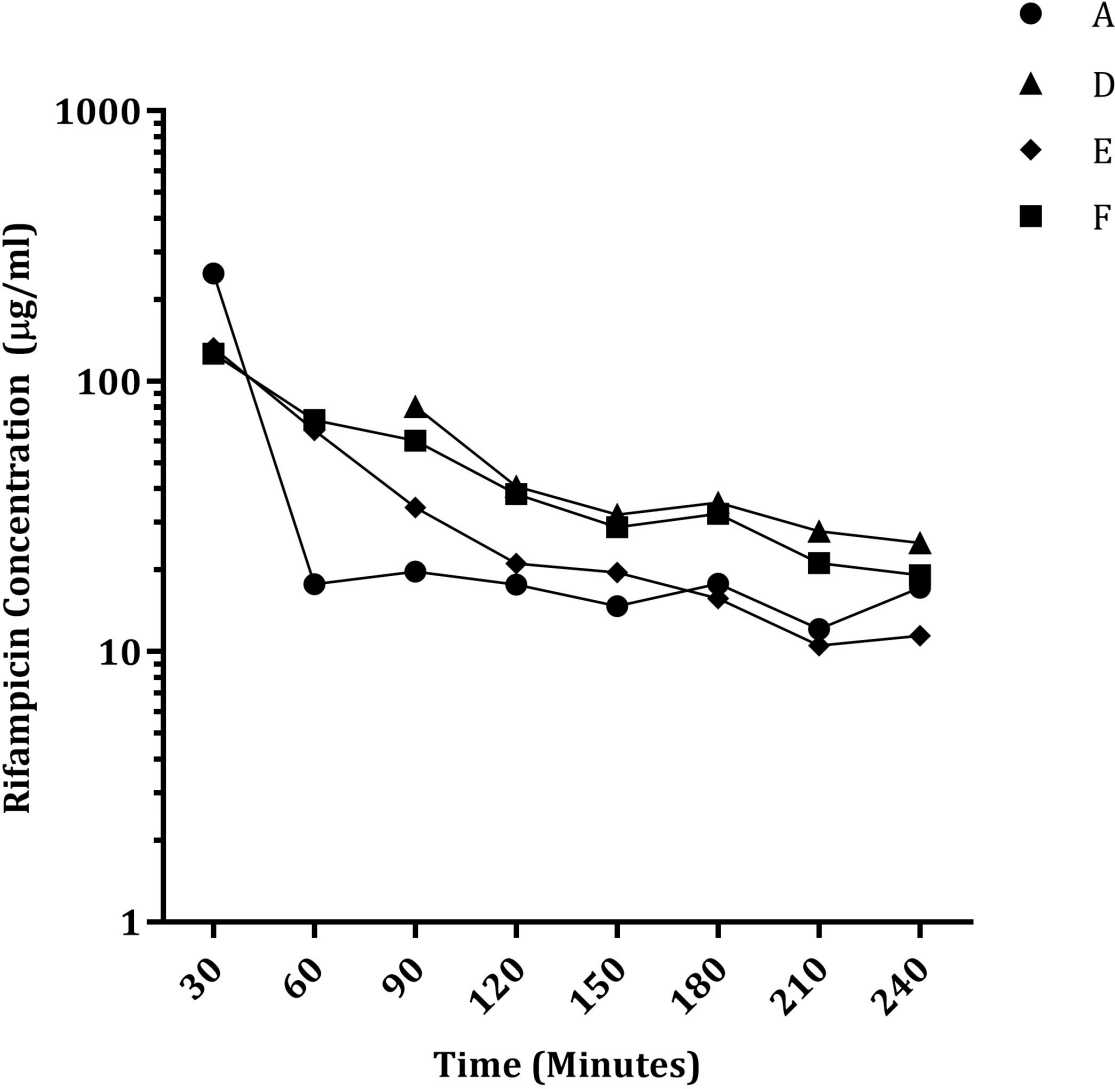
Rifampicin concentrations recovered from sacrospinalis. Fractions were collected over a four-hour (240 minute) period from four guinea pigs every 30 minutes. The measured rifampicin concentrations in each fraction are shown.

**Fig 3:**
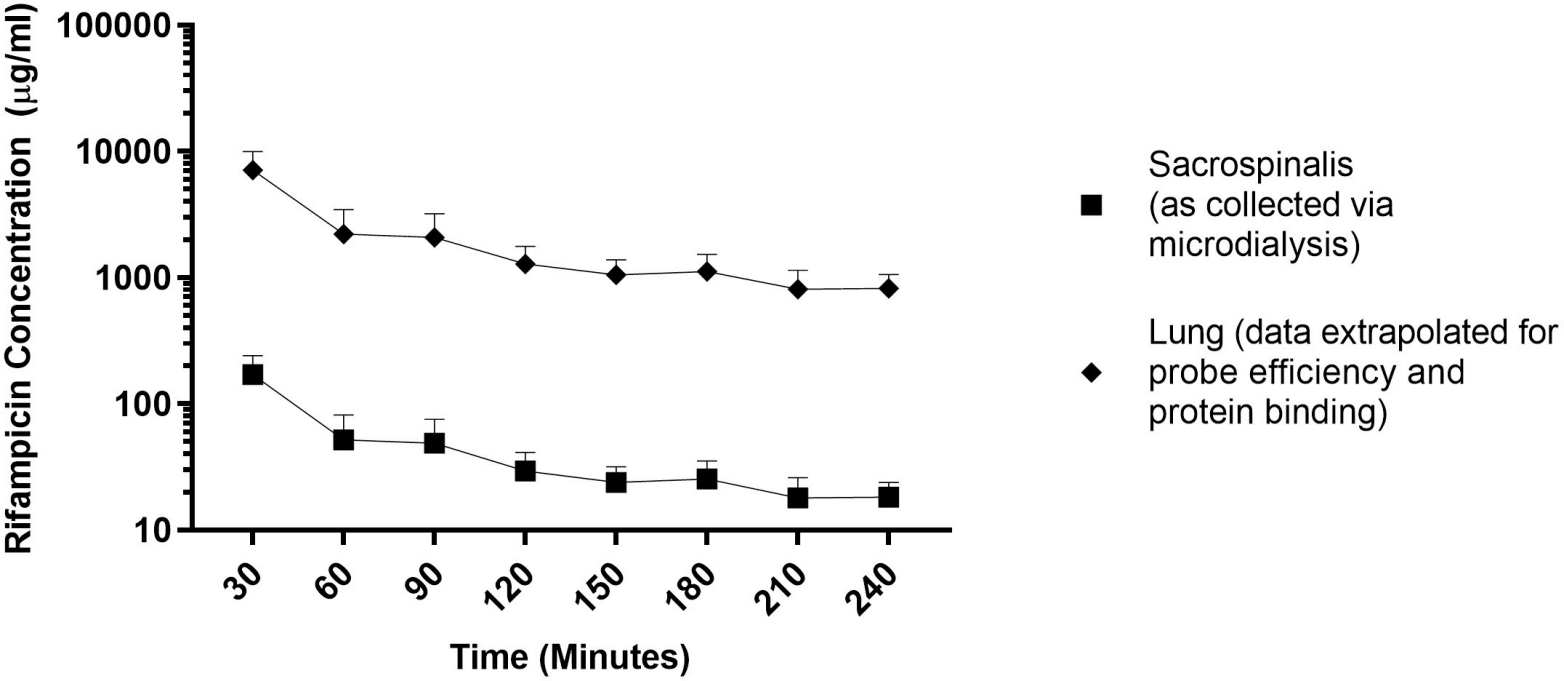
A comparison between rifampicin concentrations measured in the sacrospinalis and lung; lung concentrations were estimated from rifampicin concentrations measured via microdialysis of the sacrospinalis. Squares represent mean unbound rifampicin concentrations as recovered from the sacrospinalis via microdialysis (21% probe efficiency), diamonds are the mean total rifampicin concentrations in the lung as calculated by the equation shown in Fig 1 and corrected for 69% tissue protein binding and probe efficiency (21±9%). Error bars represent standard deviation.

Unbound (UB) rifampicin concentrations were measured and the associated kinetic profile generated, over 240 minutes, via microdialysis of the sacrospinalis of four guinea pigs (Fig 2). No samples were collected at 30 and 60 minutes post drug administration for Guinea Pig D. The probe showed patency when flushed with the rifampicin-containing solution and during the one-hour pre-drug administration flush, however no dialysate was collected. Following a rearrangement of the tubing, samples were collected from 90 minutes onwards. Samples from Guinea Pigs E and F contained the approximately the same concentrations at 30 and 60 minute time points (E; 132 and 65μg/ml F; 126 and 72μg/ml). The recovered rifampicin concentrations in Guinea Pigs D and F were very similar between 120 and 240 minutes. Whilst the kinetics (change over time) of the datasets from each of the four guinea pigs was similar, there were variations in the actual values. As mentioned, samples from Guinea Pigs D and F contained similar quantities of rifampicin between 120 and 240 minutes (D; 40, 32, 35, 27, 25μg/ml and F; 38, 28, 32, 21,19μg/ml) but these concentrations were higher than those recovered from Guinea Pigs A (17, 14, 17, 12, 17μg/ml) and E (21, 19, 15, 10, 11μg/ml) with the exception of the higher drug concentration at the 30 minute time point associated with Guinea Pig A (250μg/ml). These data were used to estimate rifampicin concentrations in the lung (using the equation Y = B0+B1*X) and an estimate of the total unbound *in vivo* rifampicin concentrations in the lung was made by correcting for probe efficiency (21±9%). A further adjustment was made to estimate the total (Bound+UB) rifampicin concentrations in the lung by correcting for protein binding (69%) (Fig 3) in order to compare these data to those measured in human as microdialysis only collects the UB portion of the drug [6].

### Preliminary drug disposition

To generate the AUC (area under the curve), rifampicin concentrations as measured via microdialysis in the sacrospinalis and correlated estimated lung rifampicin concentrations at 100% probe efficiency with and without protein binding corrections were analysed using PK Solver [18]. A one-compartment analysis was conducted using first order kinetics for each dialysed guinea pig (R^2^ = 0.979). To enable a comparison between the estimated guinea pig lung and human blood AUC_0-t_ range, these data were corrected for 69% tissue protein binding which generated a total lung AUC_0-t_ of 34.2mg/L*h.

## Discussion

Microdialysis, the only methodology available to measure unbound drug concentrations in organs of live, freely moving animals, was used to estimate rifampicin concentrations over time in guinea pigs. Dialysis of the sacrospinalis was conducted in order to estimate the unbound rifampicin concentrations in the lung and this was only possible after the identification of a correlation between rifampicin concentrations measured in the lung and sacrospinalis. The relationship between lung and skeletal muscle drug concentrations of drugs (gatifloxicin and cefpodoxime) was identified in two separate studies conducted by Liu and Tasso et al. [12, 13]. Isoniazid has similar physiochemical properties to gatifloxicin and cefpodoxime but, in our studies, no relationship was identified between lung and skeletal muscle isoniazid concentrations which was unexpected. However, a correlation between rifampicin concentrations in the lung and skeletal muscle was identified (Pearson R^2^ = 0.8595 (P value = 0.0001)) and therefore it was decided that *in vivo* microdialysis of would be trialled, using rifampicin. Guinea pigs were administered one oral dose of 50mg/kg rifampicin [8] with the aim of measuring rifampicin concentrations in the sacrospinalis over a 4 hour period post-dosing. 4 hours was chosen for the dialysis period as the Tmax and Cmax of rifampicin in a previously published study were reported as 2.0±0.01h and 2.9±2.0mg/L respectively following a single 50mg/kg dose of rifampicin [11].

The continuous serial sampling of organ drug concentrations is a key advantage of the microdialysis methodology because a more complete profile is generated of drug concentration over time in the biophase, which is more clinically relevant than the blood. It is important to mention that although the microdialysis methodology is able to collect samples more frequently, each sample, although analysed as a 30 (or 30 minute increment) minute sample, actually represents the concentration of rifampicin recovered between 0 and 30 minutes.

The estimated unbound concentration of rifampicin in the lung, as measured by microdialysis in the sacrospinalis and extrapolated using the equation determined by the correlation in Fig 1, with and without correction for probe efficiency, was higher than the total rifampicin concentration measured in the lung homogenates following necropsy (between ~1.75 to 2.5 fold higher). It could be concluded that the use of estimations based on the unbound rifampicin concentration measured via microdialysis over-represents the drug concentrations in the organs, however it could also be argued that homogenisation of the lungs and associated sample processing results in drug loss due to the lipophilic nature of rifampicin (LogP = 3.85) and the associated non-specific binding to plastics. The lipophilic nature of rifampicin was evident during the microdialysis method development where an increased relative recovery was observed associated with prior exposure of the probe to rifampicin. It is likely that the previous exposure to rifampicin, would result in saturation of binding to the surfaces, hence when the probe was re-used there were no (or minimal) binding sites for the rifampicin to non-specifically bind (adhere) to, resulting in more drug being collected in the dialysate [19]. An additional probe modification to improve relative recovery was the use of a 10mm as opposed to a 4mm membrane due to the larger membrane surface area for diffusion to occur. Choice of molecular weight cut-off had minimal effect on relative recovery, however use of the 100kDa probe would prove to be problematic when conducting microdialysis *in vivo* as in guinea pigs rifampicin is 69.5% plasma protein bound, binding to a range of proteins (Mw: ranges between 23 to 250kDa) [20] the majority of which would pass through a 100kDa cut-off semi-permeable membrane.

The unbound rifampicin AUC_0-t_ estimated in the lung following a 50mg/kg dose of rifampicin in guinea pigs was 10.62mg/L*h or 34.2mg/L*h when corrected for protein binding and probe efficiency. Estimating total unbound *in vivo* rifampicin concentrations in the lung by correcting for probe efficiency calculated an unbound AUC:MIC (PK/PD parameter associated with efficacy of rifampicin) of 237. This was close to 271 for total (B and UB) rifampicin AUC:MIC which is the figure associated with a therapeutic effect in mice [21].

Whilst the method needs to be tested with additional drugs and ultimately in infected guinea pigs, in which the skeletal muscle may not be a useful surrogate for the lung, the ability to perform the microdialysis technique in guinea pigs will be a good starting point to investigate of the kinetics of drugs over time in a more clinically relevant site than the blood. Therefore, in the future the main application of the microdialysis methodology in the guinea pig could be centred around dose optimisation studies during pre-clinical drug development with the aim to achieve appropriate AUC:MIC ratios in the lungs rather than in blood. Each new drug would require a period of *in vitro* optimisation but could then be trialled by microdialysis of guinea pigs to generate data that could be translated via the use of PBPK models into doses to begin further *in vivo* studies, especially studies that predict human pulmonary and lesion exposures [22].
